# Genomic data sharing in Europe is stumbling—Could a code of conduct prevent its fall?

**DOI:** 10.15252/emmm.201911421

**Published:** 2020-02-18

**Authors:** Fruzsina Molnár‐Gábor, Jan O Korbel

**Affiliations:** ^1^ Heidelberg Academy of Sciences and Humanities Heidelberg Germany; ^2^ European Molecular Biology Laboratory Genome Biology Unit Heidelberg Germany

**Keywords:** Chromatin, Epigenetics, Genomics & Functional Genomics, Genetics, Gene Therapy & Genetic Disease, S&S: Ethics

## Abstract

Genomic data sharing is becoming more important as scientists join forces across borders in biomedical research for the benefit of patients and society. The EU's General Data Protection Regulation (GDPR) helps simplify sharing of such data at the European and international level. However, initial optimism has dried up as EU member states go their own ways in implementing the GDPR into national laws, and as legal cases challenging data sharing reach courts. Codes of conduct could facilitate data sharing in Europe and better connect it to global health research. This commentary explains the potential of codes of conduct for addressees and drafters. Codes are no panacea though; other measures may be necessary to ensure that Europe remains collaborative and competitive in biomedical research. Nevertheless, codes of conduct would bring immediate benefits and, in the long term, could foster a true European ecosystem for joint biomedical research and easier international data sharing.

## Graphical abstract

In this commentary, F. Molnár‐Gábor and J.O. Korbel help us to understand how Codes of Conduct would facilitate the EU's General Data Protection Regulation (GDPR) in simplifying sharing of genomic data at the European and international level.

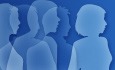

**Glossary^1^**
Adequacy decision: The European Commission can determine whether a country outside the EU/EEA has an adequate level of data protection. The level of data protection counts as adequate if it is essentially equivalent with the level of protection provided by the GDPR in the EU. The effect of such a decision is that personal data can flow from the EU to that country without any specific authorization. The European Commission has so far recognized Andorra, Argentina, Canada (commercial organizations), the Faroe Islands, Guernsey, Israel, the Isle of Man, Japan (business operators), Jersey, New Zealand, Switzerland, Uruguay, and the United States of America (limited to the Privacy Shield framework) as providing adequate protection. Adequacy talks are ongoing with South Korea. Data exchanges in the law enforcement sector are not covered by adequacy decisions, but instead by the so‐called “Police Directive” (Directive (EU) 2016/680).
Broad consent: The GDPR allows for the possibility that when scientific research involves collecting personal data, it may be impossible to fully specify the precise purposes of any data processing in advance. Therefore, data subjects should be allowed to give their consent to certain areas of scientific research when in keeping with recognised ethical standards for scientific research Member states have interpreted these GDPR rules very differently, and some data protection supervisory authorities interpret them strictly. The Article 29 Working Party has recently issued an opinion on consent, including on questions related to broad consent.
Consent: Any freely given, specific, informed, and unambiguous indication of a data subject's wishes; a subject must provide a statement or a clear affirmative action that signifies their agreement to the processing of their personal data.
Data controller: A natural or legal person, public authority, agency, or other body that—alone or jointly with others—determines the purposes and means of the processing of personal data.
Data processor: A natural or legal person, public authority, agency, or other body that processes personal data on behalf of a data controller.
Data subjects’ rights: The GDPR provides various rights to data subjects, derived from the general fundamental right to the protection of personal data. Understood as an operationalization of this general right, the most important are as follows: the right to be informed, the right of access, the right to rectification, the right to erasure, the right to restrict processing, the right to data portability, and the right to object, as well as rights in relation to automated decision‐making and profiling. These rights must be guaranteed throughout data processing. How they are safeguarded is an important cornerstone of the EU Commission's assessment of data protection levels in countries outside the EEA. In limited cases, some rights might be restricted. The GDPR allows member states to define restrictions under certain conditions.
European Data Protection Board: an independent body of the EU with its own legal personality. It consists of the national supervisory authorities of the EU and the EEA, the European Data Protection Supervisor, and the European Commission (with limited rights). It advises on the interpretation of the GDPR and works toward a standardized application of the GDPR across EU countries.
EU‐US Privacy Shield: The EU‐US Privacy Shield frameworks were designed by the United States (US) Department of Commerce and the European Commission to provide companies on both sides of the Atlantic Ocean with a mechanism that ensured compliance with data protection requirements when transferring personal data from the EU and Switzerland to the US.
GDPR: General Data Protection Regulation. This regulation sets out the rules on data protection within the EU. It entered into force in May 2016 and regulates processing of personal data relating to individuals in the EU by individuals, companies, or organizations. It does not apply to the processing of the personal data of deceased persons. Nor does it apply to data processed by an individual for purely personal reasons or for activities carried out in one's home, provided there is no connection to a professional or commercial activity.
Jurisdiction: The authority of a court or other institution to make decisions or judgments
Legal basis for data processing: The legal justification for processing personal data. The GDPR defines various legal bases such as the consent of the data donor, performance of a contract, legitimate interests, a vital interest, a legal requirement, and public interest. If the data processed are sensitive, both a lawful basis for general processing and an additional condition for processing this type of data must be identified. Some member states with civil law tradition adopt the position that a specific condition about the legal basis for processing replaces the general requirements. One such additional condition is processing for scientific research purposes.
Personal data: Any information relating to an identified or identifiable natural person, i.e., a person who can be identified, directly or indirectly, by methods including reference to an identifier such as a name, an identification number, location data or an online identifier, or reference to one or more factors specific to their physical, physiological, genetic, mental, economic, cultural, or social identity.
Pseudonymization: The processing of personal data in such a manner that the personal data can no longer be attributed to a specific data subject without the use of additional information, provided that such additional information is kept separately and is subject to technical and organizational measures that ensure that the personal data are not attributed to an identified or identifiable natural person
Safe Harbor Agreement: defunct predecessor to the EU‐US Privacy Shield. The EU Data Protection Directive, the precursor of the GDPR, prohibited the transfer of personal data to non‐EU countries that do not meet the EU adequacy standard for data protection. In order to bridge differences in data protection approaches and provide a streamlined means for US organizations to comply with the Directive, the US Department of Commerce—in consultation with the European Commission—developed the Safe Harbor framework to provide the information necessary to evaluate and then join the US‐EU Safe Harbor program. On October 6, 2015, the European Court of Justice issued a judgment declaring as invalid the EC's Decision 2000/520/EC of July 26, 2000 “on the adequacy of the protection provided by the safe harbour privacy principles and related frequently asked questions issued by the US Department of Commerce”.
Standard contractual clauses: The European Commission can decide that standard contractual clauses (SCCs) offer sufficient safeguards on data protection for the data to be transferred internationally. It has so far issued two sets of SCCs for data transfers from data controllers in the EU to data controllers established outside the EU or EEA, and one set of SCCs for data transfers from controllers in the EU to processors established outside the EU or EEA.
The Court of Justice of the European Union: the principal judicial institution of the EU and its highest court. It currently consists of one judge from each member state and eleven advocates general. The European Commission, or another member state, may bring an action before the Court of Justice against a member state on the grounds of a failure to fulfill an obligation under the EU treaties. The European Court of Justice is part of The Court of Justice of the European Union.

## Data sharing: the state of the art

Cross‐border data sharing has become increasingly important in genomics, as exemplified by the launch of many large‐scale multinational research projects during the past two decades. One such project is the International Cancer Genome Consortium (ICGC), and its Pan‐Cancer Analysis of Whole Genomes initiative (PCAWG) and Accelerating Research in Genomic Oncology project (ICGC‐ARGO) rely on cross‐continental sharing of cancer genomes and transcriptomes (Stein *et al*, 2015; PCAWG consortium, 2020). Other projects focus on rare disease studies such as RD‐Connect, which benefits from coordinated access to genomic and phenotypic information from individuals with unexplained diseases (Lochmüller *et al*, 2018, cf. *Glossary*). In basic research, the Human Cell Atlas relies on data sharing to create reference maps of all human cell types to further research into human biology and disease (Regev *et al*, 2017).

Research with ‐omics data, whether from genomes, transcriptomes, epigenomes, or metagenomes, requires establishment of and access to large community reference data collections. These are often generated in the context of international consortia. External datasets are also necessary to replicate and verify discoveries. These reference collections and datasets might be in another country or another continent. The distinctiveness and diversity of rare diseases and different types of cancer, combined with the small number of patients for many disorders, not only effectively precludes conventional research discovery based on local sample cohorts, but also mandates cross‐matching data between centers to increase cohort size and enable discoveries, replication, and translation of findings into therapies.

The Global Alliance for Genomics and Health (GA4GH), an organization responsible for framing policy and setting technical standards, has estimated that by the end of 2021 around 50 million human genomes or exomes will have been analyzed via whole genome and exome sequencing, with much of the data coming from health care (preprint: Birney *et al*, 2017). To maximize the value of this information for disease diagnosis and to enable translation into practical medicine, institutions need to be able to share these data across borders. Currently emerging projects, such as an initiative to provide access to at least 1 million human genomes in Europe by 2022, rely on large‐scale sharing of patients’ genomic data, as well as other sensitive information, across numerous countries (Saunders *et al*, 2019). Genomic and health‐related data being shared not only count as personal data (cf. *Glossary*) in that they may allow donor re‐identification, but are also, by their nature, considered as particularly sensitive in relation to the fundamental rights and freedoms of sample donors. According to the majority of data protection laws, such data merit specific protection as its processing could pose significant risks to those rights and freedoms (Knoppers *et al*, 2018).

Data sharing across borders has been transformative for research on both rare diseases and cancer. Each individual rare disease is so scarce that individual centers and often entire countries may lack the patient cohorts to meaningfully interpret the disease. Cumulatively, however, 4% of European children have a rare disease, which highlights the potentially transformative effect of sharing data across borders to increase sample sizes to a level that makes investigation of each individual disorder feasible. A similar situation exists in the context of cancer, where individual cancer types or subtypes could be considered a “rare disease”, the investigation of which is made vastly easier by data sharing. One example is research on childhood medulloblastoma, where cross‐border sharing of patient genetic and clinical data within Europe and beyond led to breakthroughs that uncovered the frequent hereditary basis of the disease and led to new recommendations for clinical management (Waszak *et al*, 2018, 2020; Begemann *et al*, 2020). However, despite the clear need to share data in translational genomic research, options for doing so legally are becoming alarmingly cumbersome—which may ultimately hamper biomedical and genomic research in Europe or with European participation.

The EU General Data Protection Regulation (GDPR, cf. *Glossary*), which entered into force in May 2016, aims to secure a high level of protection of personal data in all EU member states. Data can cross borders within the European Economic Area (EEA) if the planned processing complies with the general requirements of the GDPR. Generally speaking, the fact alone that data cross borders does not require the fulfillment of legal requirements beyond those placed on the processing itself. Nevertheless, the requirements of applicable member state laws must also be fulfilled in addition to the GDPR.

If data are transferred outside the EU, the planned processing must not only comply with the general requirements of the GDPR and relevant member state provisions but must also meet the conditions for transferring genomic data outside the EU as defined by the GDPR. These conditions, any one of which must be fulfilled to gain permission for data transfer, ensure that the level of protection cannot fall below the level guaranteed by the GDPR itself, even if data are transferred outside the EU.

## Uncertainty around data transfers within the EU

Even though the GDPR aims to enable the free flow of data within the EU, harmonization is still lacking in many areas because the GDPR gives member states extensive room to implement their own rules, especially for the processing of genomic and health data. Even though these rules should not interfere with the free cross‐border movement of data, member states may deviate “upwards” from the GDPR's level of data protection and specifically restrict processing of such data.

Furthermore, this room for national implementation includes latitude to design rules related to the processing of sensitive data for scientific research purposes. It is generally allowed under specific conditions, such as appropriate safeguards to guarantee data subjects’ rights, and when it is explicitly justified by research. Such processing must also be based on EU or member state law. However, the GDPR provides no closed rules for member states regarding the concrete design of appropriate safeguards, which primarily should guarantee that technical and organizational measures are in place to ensure respect for the principle of data minimization. Furthermore, member states can define derogations from some of the data subjects’ rights in such processing contexts under certain conditions (the rights of access, rectification, restriction, and erasure as well as the right to object), and many have done so. In the Netherlands, for example, research institutions may choose not to apply rights of access, rectification, and restriction—provided they ensure that personal data can only be used for statistical or scientific purposes. In Germany, for example, it must remain impossible to draw direct conclusions about specific individuals. In the UK, results must not be made available in identifiable form (Boardman & Molnár‐Gábor, 2019).

Finally, the lack of harmonization of data protection rules across Europe must also be kept in mind, as said rules may influence data processing for scientific research purposes, such as determining conditions under which processing personal data can generally be lawful. Many member states have written their own rules on the role of consent—especially broad consent (see *Glossary*)—for the processing of genetic and health data or may in the future specifically define what exactly constitutes “public interest”, which could also influence the lawfulness of processing for scientific research purposes. If multiple research stakeholders within the EU work together to process data, or a single stakeholder operates in multiple EU countries, identifying a (common) justification for processing personal or even sensitive data is challenging.

One potential solution would be to avoid dealing with personal data at all. Unfortunately, data used in genomic research are by necessity personal and sensitive, as samples can unambiguously be traced back to an individual with the help of only around 10 single nucleotide polymorphisms (SNPs). Pseudonymization has its limitations (Gymrek *et al*, 2013; cf. *Glossary*), and developments in machine learning and artificial intelligence already allow re‐identification of even small samples from anonymized data sets (Rocher *et al*, 2019). The likelihood of individual re‐identification from genomic data, whether coded or anonymized, is higher when such data have been linked with familial, sociodemographic, or audio‐visual information, as is often the case in rare diseases research (Thu Nguyen *et al*, 2019). Finally, any data encoding or security measure has to accept that research results will hopefully be transferred back into healthcare settings. Therefore, further standardization of how personal data are shared within EU borders and beyond would certainly be advisable.

## International data sharing at the precipice

Sharing data with third countries, including Canada and the USA, is arguably crucial for research and translation. One could envision a possible scenario where European data are to be uploaded into a databank located in the USA and operated by a US provider, which would apply internationally available “controlled‐access” principles to data in disease research.

In order to transfer personal and sensitive data to countries outside the EU, special arrangements must be made if the destination country does not provide an adequate level of data protection. The adequacy (see *Glossary*) of the level of data protection in the respective country is assessed by the European Commission, which requires an extensive investigation into relevant laws and rules including data protection supervision and redress for data breaches. There are currently only 13 countries worldwide that fulfill this criterion. The Commission's decision on the adequacy of data protection in the relevant country then has effect in the entire EU.

Where adequacy has been established, transfers of personal data to that country may take place without the need to obtain any specific authorization. Countries covered by an adequacy decision include Japan (for transfers to business operators), Argentina, Israel, Switzerland, and Canada (for commercial entities), and the USA (limited to the Privacy Shield framework). Until now, researchers’ and their institutions’ best bet was thus to rely on the Commission's adequacy decision that the level of personal data protection is essentially equivalent with that of the GDPR. Regarding transatlantic data transfers, the Privacy Shield agreement made between the EU Commission and the US Department of Commerce served as the basis of the EU adequacy decision, because it imposes specific rules on the participating US entities to better protect the data of EU citizens compared to the data protection laws generally existing in the country (cf. *Glossary*).

If there is no adequacy decision from the Commission with regard to a specific country, data may be transferred if the participating actors—those that control data processing and those that actually process data (cf. *Glossary*)—have provided appropriate safeguards, and on the condition that enforceable rights and effective legal remedies for data subjects are in place. Appropriate safeguards have until now probably best been provided by adhering to so‐called standard contract clauses (SCCs, cf. *Glossary*), a standard set of contractual terms and conditions issued by the Commission to protect personal data leaving the EU. Both the sender and the receiver sign up to the contractual obligations. While the SCCs release the contracting parties from having to negotiate individual terms, the parties can include other safeguards or additional clauses. However, the SCCs have to be adopted in a complete and unaltered manner and additional clauses added by the parties cannot contravene the SCCs, as the Commission has declared those very model rules as being in compliance with the GDPR.

While the Privacy Shield and the respective adequacy decision of the EU Commission only allow European data transfers into the USA and come with certification obligations for data recipients, SCCs can be used for relatively straightforward international data transfers to any country in the world.

Currently, cases are being heard by the European Court of Justice regarding both SCCs (ECJ, C‐311/18) and the Privacy Shield (ECJ, T‐738/16) (ECJ, see *Glossary*). Complaints against the Privacy Shield emphasize that it does not prevent the processing of EU citizens’ personal data by US surveillance authorities and, thus, does not provide an adequate level of data protection. This was already a key reason for the annulment of its predecessor, the Safe Harbor scheme, by the ECJ (cf. *Glossary*). Similar concerns have been raised about SCCs, but the key questions focus on whether personal data transferred under SCCs will be subject to an adequate level of protection purely by virtue of entering into SCCs, or whether the legal system in the recipient country should also be analyzed.

Advocate general of the ECJ Henrik Saugmandsgaard Øe delivered his opinion on SCCs in mid‐December 2019. In his view, SCCs provide a general mechanism for data transfers irrespective of the country of destination and the level of protection guaranteed there. The validity of SCCs depends only on the safeguards they provide in order to compensate for any inadequacy of data protection in the destination country. However, there should be an obligation—placed on the data controllers (cf. *Glossary*) and the supervisory authorities—to ensure that transfers based on SCCs are suspended or prohibited whenever those clauses are breached or impossible to honor. This means that the level of protection for the transferred data can only be assessed on a case‐by‐case basis.

Naturally, the general level of data protection in the destination country is decisive for determining whether the SCCs can be honored and applied effectively. Thus, the significance of the second case before the ECJ—dealing with the Privacy Shield and thus with the laws and practices in the USA in general—will go beyond the scope of data transfers based on the EU Commission's adequacy decision as it is irrespective of the legal basis of the transfer, whether this be SCCs or codes of conduct. According to the general attorney's opinion, this does however not hinder supervisory authorities’ investigations into the law of the destination country, nor does it pre‐empt their decision on a transfer when relying on a legal basis for the transfer other than the adequacy decision itself.

In the meantime, important changes related to data protection and especially supervision have been pushed forward in the USA. It remains to be seen whether and how these changes influence the ECJ's evaluation of their level of data protection. However, if the ECJ strikes down SCCs in their entirety, it would impede not only transatlantic data sharing but *all* data sharing with non‐EEA countries. This could leave Europe's researchers in a legal quagmire regarding data sharing with global partners as early as this year. Another possible outcome is that the ECJ upholds the validity of SCCs, but the competent supervisory authority could still suspend individual data transfer into the USA. However, such an outcome—even if due to other reasons than the alleged inadequacy of data protection in the USA—might well influence supervisory authorities’ general practice, as these authorities gradually gain practical experience and exchange information with each other within the EU.

In the absence of both an adequacy decision and appropriate safeguards, data transfers could still be based on certain derogations. The most obvious would be the data donors’ explicit consent (see *Glossary*). However, this may, especially in the case of retrospective data analyses, be missing or might not cover the particular processing in question. Also, a relevant supervisory authority's authorization for specific data transfers will usually follow a lengthy and cumbersome application process, thus hindering smooth international data flows. Last but not least, in the absence of an adequacy decision, member state law may, for important reasons of public interest, expressly set limits on the transfer of specific personal data to a third country. Although most member states have not implemented any restrictions beyond those set out in the GDPR, Denmark and Cyprus, under certain conditions, apply additional restrictions to the transfer of sensitive personal data to third countries.

## Codes of conduct: false short‐term expectations

As specific data processing fields can have particular needs, the GDPR allows establishment of sector‐specific rules in the form of codes of conduct (Philips *et al*, 2020). Such codes are being developed both within the EU and internationally, and the hope is that they could lead to greater standardization within the EU and easier international data transfers.

In early summer 2019, the European Data Protection Board (EDPB, see *Glossary*) adopted Guidelines on Codes of Conduct (EDBP). The aim of these guidelines is to provide practical guidance and assistance to clarify the procedures and rules involved in the submission, approval, and publication of, and the monitoring of adherence to, codes of conduct at both the national and the European level. In the meantime, many codes have emerged or are being drafted. An initiative led by the Biobanking and BioMolecular Research Infrastructure BBMRI‐ERIC is aiming to create a GDPR‐based code of conduct for health research data processing within the EU (BBMRI).

However, there are some false expectations; in particular, codes of conduct cannot directly provide for an immediate increase in the harmonization of regulations on data processing within the EU, at least in the short term. First, a code of conduct can only specify data processing rules within the limits set by the GDPR. Second, EU law—and thus the Commission's decision that an approved code of conduct has general validity within the EU—has priority over member state laws. When applying the GDPR, this priority only pertains to the extent that member state law contradicts EU law. That member states will derogate from the GDPR in their implementation is foreseen in the GDPR itself. Even strict member state regulations on the processing of genomic data are enabled by the GDPR and thus would not contradict it, despite its general slant in favor of the free flow of data. Third, the Commission's decision to approve a code has to be consistent with the GDPR, including allowing member states to derogate from its rules.

The overall consequence is that codes of conduct can—by distilling and clarifying GDPR rules—provide for further harmonized interpretation, but only of those rules that cannot be subject to derogation by member states, or where member states did not choose to derogate. In the case of data processing for scientific research, this will, for instance, exclude interpretation of important rights of the data subjects such as the right to information, the right to rectification, the right to restriction of processing, and even the right to object. Altogether, given that member states have nearly finalized their GDPR implementations, there is indeed little room left for further harmonized interpretation through a code.

Even if we presume that the implementation of rights related to data protection for processing in research health contexts might be better clarified through a code at the international level, those rights must also be effectively claimed by data subjects. Also, when it comes to the specific issues currently before the ECJ, codes do not offer any better solution in the short term than the Privacy Shield or SCCs as legal challenges are primarily related to the level of data protection in the respective country. At the very least, legal protection could be sought against unlawful or inadequately enforced codes that have general validity in the EU, if data processors have bound themselves legally *vis‐á‐vis* the treatment of data subjects. Namely, data recipients outside the EEA would still have to make binding and enforceable commitments in order to be covered by the code. Legal challenges—including jurisdictional matters—related to the assessment and fulfillment of such commitments also need to be carefully considered, taking into account specific aspects of data processing. The decisions the ECJ will make in the pending cases, particularly on SCCs, will thus be instructive and might contribute to clarifying the conditions under which recipients in countries outside the EEA might adhere to a code of conduct.

## Long‐term benefits of codes of conduct

Given all the above, is it still worth working on a code of conduct for health research? There are indeed some benefits: notably, codes of conduct can be written with the help of researchers. Their participation increases the chance that data protection issues will be addressed according to the needs of the relevant sector, that is, genomic and health research. Equally, their involvement can help to increase the code's acceptance: Legally speaking, scientists’ participation can strengthen a code's factual legitimacy. This is particularly important if codes are to become a benchmark for “reasonable care”, which can have significant implications on how liability is assigned and acknowledged [compare Table 1]. Such actual compliance with state‐of‐the‐art scientific practice is independent of an approval of a code of conduct by the Commission and could be initiated by a voluntarily adopted code. Thus, a code of conduct can also represent an important step toward further codification and integration of research ethics standards, presumably as appropriate safeguards within data protection law.

**Table 1:** Codes of conduct (CoCs)

5 important points on scientific self‐regulation
1. Rulemaking on data processing in a specific field based on a bottom‐up approach
A CoC on health research could be (co‐)developed with help from scientists
2. Various forms of CoCs exist: pure or private self‐regulation, regulated self‐regulation, or co‐regulation
The GDPR implements regulated self‐regulation as it provides a legal framework for the establishment, approval and monitoring of CoCs
3. A CoC is based on the sectoral and disciplinary expertise of the involved drafters
The GDPR does not define a closed list of rules that can be included in a CoC, so a CoC can also integrate ethical principles and technological knowledge.
4. For general legal relevance, the legislator must require compliance with the rules of a CoC
The GDPR provides for the approval of CoCs by the Commission such that they then become generally binding in the EU
5. A Code of Conduct can influence formal laws
A CoC can contribute to the sectoral standardization of data processing in a scientific research field, positively influencing regulatory decisions on permits and reservations.

In addition to member states’ implementations of the GDPR, the applications of rules in the GDPR that are directly binding and cannot be implemented differently can still be clarified via a code. Moreover, regulatory gaps in the GDPR can be closed by a code in a manner compliant with the GDPR, as the practice around the GDPR is still in its infancy. Altogether, this could lead to more consistent application of laws by supervisory authorities, even across borders in certain cases. Through the back door of application, a code could, in the long term, also influence member states’ regulatory approaches, which could lead to closer, more coordinated understanding of the GDPR's rules in different EU countries. In return, however, any code of conduct for health research can only increase its impact in better enabling EU‐wide data sharing if member state implementations of the GDPR are closely analyzed in advance and are taken into account when drafting the code. Awkward as it may seem, this research needs to be done before drafting of any code of conduct can begin.

The GDPR provides incentives for developing codes of conduct in the form of privileges for those responsible for data processing. One such privilege is being able to draw on a code to demonstrate the fulfillment of data protection duties and security measures, thus setting appropriate safeguards that those processing data and those controlling this processing could adhere to. Thus, a code can contribute to harmonization of data protection obligations, which can enhance legal certainty for data donors and lead to a common understanding of justified and standardized expectations regarding safeguarding and implementing donors’ data protection rights in various processing situations.

## Discussion

We have highlighted several emerging legal issues on the European and international level that might negatively influence data sharing in genomic research. One way to at least mitigate legal challenges might be the establishment of a code of conduct. In the short term, however, a code cannot force direct further harmonization of EU member state data protection rules or immediate circumvention of long‐standing issues in international data sharing. A code of conduct also cannot release policymakers or researchers (and the legal departments of their institutions) from the burden of negotiating and drafting the legal documents needed to support and supplement such a code under the law to enable data processing and transfer.

However, self‐regulatory rulemaking, as well as clarification of data protection rules and the specification thereof, can grant a code of conduct important legal relevance both within the EU and internationally: immediately, by easing responsibility issues through clarifying appropriate safeguards for data protection, in the medium term, by promoting coordinated rule application, and in the long term, by influencing laws that directly regulate data sharing through enhancing a common understanding of data protection obligations. These benefits make codes of conducts initially stand out from other measures and eventually might enable them to provide solutions for longer‐term challenges. They could thus contribute enormously to the clarification of daily data processing challenges, such as under which conditions the presumption that further processing for scientific research purposes is compatible with the initial purpose is applicable.

Put simply: A code of conduct can help standardize rules for scientific data processing. The standardizing effect, however, can only be sectoral. It must therefore be recognized that codes of conduct could pave the way for a further—not national but sectoral—diversification of data protection. This tendency of data protection development is ultimately inevitable, as data are considered personal in specific processing contexts and related to particular processing methods, and privacy and the right to the protection of personal data are defined, as ever, in a contextualized manner.

Moreover, a code of conduct represents a positive approach, generating rules to allow data processing for research. As such, even established within the realm of the GDPR, they stand in contrast to the EU's current regulatory approach exemplified by the GDPR, in which data processing is generally banned apart from specific exceptions. As it specifies the exception in the interest of scientific research and generates rules *for* data processing, it could be a milestone on the way toward shifting the overall focus of the regulatory approach to one of the general permissions in contexts such as genomic research, an area in which benefits for patients and society are so overwhelming that specific bans for particular processing activities should perfectly suffice to hinder abuse without hindering researchers’ ability to make those benefits a reality.

## Conflict of interest

FMG is currently contributing to the BBMRI‐ERIC drafting GDPR Article 40 code of conduct working party. This paper is written in a purely private capacity, and the views expressed here cannot be attributed to anyone other than the authors.

## Footnotes

1 Correction added on 6 March 2020, after first online publication: the Glossary has been updated.
